# LINC01535 promotes hepatocellular carcinoma proliferation and metastasis by regulating the miR-214-3p/VASP axis

**DOI:** 10.7150/jca.91756

**Published:** 2024-05-20

**Authors:** Chunjiang Liu, Kuan Li, Wenzhou Ding, Xiaoqi Tang, Zhifeng Wu, Xin Zhu, Wanwan Gong, Hui Zhao

**Affiliations:** 1 Department of General Surgery, Shaoxing People's Hospital, Shaoxing, 312000, China.; 2 Department of Hepatobiliary Surgery, Kunshan Hospital of Traditional Chinese Medicine, Suzhou, 215000, China.; 3 Hepatobiliary Center, The First Affiliated Hospital of Nanjing Medical University, Nanjing, 210000, China.; 4 Department of Hepatopancreatobiliary Surgery, Jiangnan University Medical Center, Wuxi, 214002, China.

**Keywords:** LINC01535, miR-214-3p, VASP, HCC, proliferation, metastasis

## Abstract

**Background:** Emerging evidence has indicated that long noncoding RNAs (lncRNAs) are associated with the development and progression of several carcinomas, including hepatocellular carcinoma (HCC). However, the role of LINC01535 in HCC is still unknown.

**Materials and methods:** In this study, RNA-seq, CCK-8, colony formation, wound healing, Transwell and tumor xenograft assays were used to explore the function of LINC01535 in the proliferation and metastasis of HCC *in vitro* and *in vivo*. Fluorescence *in situ* hybridization (FISH) assay, bioinformatics analysis, dual-luciferase assay, quantitative real-time polymerase chain reaction (qRT-PCR), and western blot analysis were used to reveal the interactions of LINC01535, miR-214-3p and VASP.

**Results:** LINC01535 was overexpressed in HCC tissues and HCC cell lines. Gain- and loss-of-function studies revealed that LINC01535 could promote HCC cell proliferation, migration and invasion both *in vitro* and *in vivo*. In addition, upregulation of LINC01535 significantly decreased the expression of microRNA-214-3p (miR-214-3p), which was found closely associated with suppressing tumor progression. Moreover, VASP was identified as a direct downstream target gene of miR-214-3p. LINC01535 positively regulated VASP expression by sponging miR-214-3p, and VASP overexpression activated the PI3K/AKT signaling pathway and stimulated epithelial-to-mesenchymal transition (EMT) in HCC.

**Conclusions:** Our study first found that LINC01535 promoted HCC progression by regulating its downstream target, the miR-214-3p/VASP axis, via the PI3K/AKT signaling pathway. The function and novel regulatory mechanism of LINC01535 may provide a valuable target for the diagnosis and treatment of HCC patients.

## Introduction

Hepatocellular carcinoma (HCC) is a primary liver cancer that accounts for the majority of cases among different types of liver cancers. It is currently the fifth most common cancer worldwide and the third leading cause of cancer-related death. The prevalence of HCC is particularly high in China due to the incidence of chronic hepatitis B virus infection 1, 2. Due to the characteristics of vague symptoms in the early stage, strong invasiveness and high incidence of metastasis, the overall 5-year survival rate of HCC patients is still poor 3. Although treatments for HCC have improved over the past few decades, liver transplantation and hepatectomy are still the main radical treatments for HCC patients. Hepatocarcinogenesis is generally considered a complex process involving multiple genes and genetic alterations, and molecular targeted therapy for HCC is a new research hotspot 4. The lack of understanding regarding the molecular mechanisms driving HCC progression and metastasis hinders the development of effective diagnostic methods and targeted therapies. Therefore, there is a pressing need to investigate and elucidate the molecular mechanisms underlying HCC to improve patient outcomes.

Long noncoding RNAs (lncRNAs) are non-protein-coding RNA transcripts with lengths of over 200 nucleotides 5, 6. Numerous previous studies have shown that dysregulated lncRNA expression is associated with many tumor biological behaviors such as proliferation and metastasis 7. In recent years, an increasing body of research has demonstrated the involvement of lncRNAs in HCC 8. The main biological mechanisms regulated by lncRNA function include cell cycle regulation, gene imprinting, splicing regulation, translation regulation and mRNA degradation 9. LINC01535 is a newly discovered lncRNA. Existing reports show that LINC01535 expression is dysregulated and that LINC01535 acts as an oncogene in various cancers, such as cervical cancer, osteosarcoma and esophageal squamous cell cancer 10-13. However, the expression pattern, function and molecular mechanism of LINC01535 in HCC are still unknown, and need further research.

The competing endogenous RNA (ceRNA) function has been a relatively well-studied mechanism of action of lncRNAs in recent years. Through miRNA response elements (MREs), lncRNAs competitively bind to target miRNAs and further regulate the expression of target mRNAs 4, 14. miRNAs are a class of short noncoding RNAs with lengths of 21-25 nucleotides. By binding to 3'-untranslated regions (3'-UTRs) of target gene mRNAs, miRNAs cause mRNAs to be translationally repressed or degraded 15. Previous studies have demonstrated the important functions of miRNAs in various biological processes, such as cell proliferation, apoptosis, metastasis, differentiation and metabolism 16, 17. The ratios of hsa-miR-21-5p/hsa-miR-199a-5p and hsa-miR-155-5p/hsa-miR-199a-5p hold as potential molecular markers for AFP-negative HCC patients 18. Whether LINC01535 is involved in the progression of HCC by sponging miRNAs has not been investigated.

This study aimed to investigate the role of LINC01535 in the progression of HCC through *in vitro* and *in vivo* experiments. In this study, we first assessed the differences in the lncRNA expression profiles between HCC cancer and paracancerous tissues. The upregulation of LINC01535 was found to promote the proliferation and metastasis of HCC *in vitro* and *in vivo*. Furthermore, mechanistic analysis illustrated that LINC01535 functioned as a miRNA sponge to positively regulate VASP expression by sponging miR-214-3p. The upregulation of VASP activated the PI3K/AKT signaling pathway and promoted epithelial-mesenchymal transition (EMT). As far as our knowledge extends, we have discovered for the first time that LINC01535 play a pivotal role in the progression of HCC through the miR-214-3p/VASP axis. These novel findings contribute to a deeper understanding of HCC pathogenesis and open new avenues for potential therapeutic interventions.

## Materials and methods

### Rationale for performing the study

Hepatocarcinogenesis is generally considered a complex process involving multiple genes and genetic alterations. We first detected HCC and paracancer differentially expressed genes by gene sequencing, focusing on the up-regulated expression of LINC01535 in HCC, which may promote the progression of HCC. Subsequently, *in vitro* and *in vivo* experiments were designed to verify that LINC01535 can promote the proliferation and metastasis of liver cancer cells. Further designed molecular experiments revealed that LINC01535 promotes HCC progression by regulating the miR-214-3p/VASP/PI3k-AKT axis.

### Aim and objectives

Aim: The aim of the study was to investigate the role of LINC01535 in the progression of HCC through *in vitro* and *in vivo* experiments. Objectives: To evaluate the differential expression of LINC01535 between HCC cancer tissues and adjacent nontumor tissues and its association with overall survival; To investigate the function of LINC01535 in promoting the proliferation and metastasis of HCC both *in vitro* and *in vivo*. To investigate the interactions between LINC01535, miRNA, and mRNA, with a specific focus on the ceRNA function. To investigate the activation of the PI3K/AKT signaling pathway and the stimulation of EMT as downstream consequences of LINC01535/miRNA/ mRNA interactions.

### Tissue specimens

A total of 70 paired HCC and adjacent normal tissues were obtained from patients undergoing surgical resection at Shaoxing People's Hospital between October 2012 and July 2014. The inclusion criteria included:1) Accurate pathological diagnosis of primary hepatocellular carcinoma, adjacent normal tissues were ≥2 cm away from the edge of the tumors; 2) Availability of comprehensive clinicopathological data and overall survival information; 3) Mainly underwent surgical resection; 4) Willing to sign informed consent. The exclusion criteria included: 1) Perioperative death; 2) Underwent palliative surgical intervention; 3) Received treatment with other antitumor therapy; 4) Concurrently diagnosed with other malignancies. The study was approved by the Ethics Committee of Shaoxing People's Hospital (Ethics number:2022-K-Y-053-01).

### Cell culture and cell transfection

HCC cell lines (HCCLM3, Hep3B, Focus, HepG2 and SMMC7721) and immortalized human hepatocyte L02 cells were obtained from the China Center for Type Culture Collection (Wuhan, China). YY8103 cells were obtained from the Key Laboratory on Living Donor Liver Transplantation. The cells were maintained in Dulbecco's modified Eagle's medium (DMEM) (Invitrogen, Thermo Fisher Scientific, Inc., Carlsbad, CA, USA) supplemented with 10% fetal bovine serum (FBS) (Gibco, Life Technologies, Carlsbad, CA, USA), 50 U/ml penicillin (Invitrogen) and 50 U/ml streptomycin (Invitrogen) in a humidified atmosphere at 37°C with 5% CO_2_. For cell transfection, lentiviruses overexpressing LINC01535 and VASP, lentiviral-based small hairpin RNA (shRNA) against LINC01535 and VASP, and negative controls (NC) were purchased from GenePharma Co. (Shanghai, China). The miR-214-3p inhibitor (miR-214-3p-inh.), negative control (inhibitor-NC), miR-214-3p mimic and mimic NC were purchased from RiboBio (Guangzhou, China). The cell transfection assay was conducted based on the manufacturer's protocol. To construct stably expressed cells, the transfected cell culture medium was supplemented with puromycin (5 μg/mL) at 48 h after infection. The cells were selected with puromycin for approximately 2 weeks to establish stable cell clones.

### RNA sequencing

Three paired HCC and adjacent nontumor tissues were lysed by TRIzol reagent (Invitrogen), and total RNA was extracted following the manufacturer's protocol. Next, RNA sequencing and sequencing data analysis were performed by JI GUANG Gene (Nanjing, Jiangsu, China).

### Quantitative reverse transcriptase polymerase chain reaction (qRT-PCR)

Total RNA was extracted from fresh HCC tissue samples and cell lines using TRIzol reagent (Invitrogen) in accordance with the manufacturer's instructions. The concentration and purity of the RNAs were quantified by a NanoDrop 2000 spectrophotometer (NannoDrop Technologies, MA, USA). Reverse transcription was performed using the Prime Script RT reagent Kit (TaKaRa, Dalian, China). qRT-PCR assay was conducted in an ABI 7900 PCR system (ABI, CA, USA) using SYBR Premix ExTaq II (TaKaRa). For miRNA, the Bulge-Loop^TM^ miRNA reverse transcription kit (RiboBio) and the Bulge-Loop^TM^ miRNA qRT-PCR Starter Kit (RiboBio) were used. The results were calculated using 2^-ΔΔct^ method. GAPDH and U6 were used as the internal controls for mRNAs and miRNAs respectively.

### Cell viability assay

Cells (1 × 10^3^ cells per well) were seeded into 96-well plates in 100 μL culture medium. Then, 10 μL cell counting Kit-8 (CCK-8) solution (Dojindo, Laboratories, Kumamoto, Japan) was added to each well. After incubation at 37°C for 2 h, the absorbance at the 450 nm wavelength was detected using a microplate reader (Thermo Scientific, USA).

### Colony formation assay

Cells were seeded into 6-well plates at a density of 500 cells per well and incubated in DMEM with 10% FBS for 2 weeks. Then, the colonies that formed were washed with ice-cold PBS, fixed in 4% paraformaldehyde and stained with 1% crystal violet. The numbers of colonies were calculated to evaluate cell proliferation.

### Wound healing assay

The transfected cells were seeded into 6-well plates and incubated in DMEM supplemented with 10% FBS. When the cells reached approximately 95% confluence, the wells were scratched using a 200 μL plastic pipette tip. The cells were further cultured in DMEM supplemented with 1% FBS. The scratches were photographed at 0 h and 48 h using an inverted microscope (Olympus, Tokyo, Japan). Cell mobility was calculated in three different regions for statistical analysis.

### Transwell assays

HCC cell migration and invasion were assessed with Transwell chambers (Corning, NY, USA). In migration assays, 2×10^4^ cells were added to the upper chambers in 250 μL FBS-free DMEM, and 750 μL DMEM containing 10% FBS was added to the lower chambers. In invasion assays, the upper chambers were precoated with 50 μL of a 1:8 mixture of BD Matrigel (BD Biosciences, Franklin Lakes, NJ, USA). A total of 5 × 10^4^ cells in 250 μL of FBS-free DMEM were added to the upper champers, and the other steps were the same as those for the migration assays. Forty-eight hours later, the cells on the upper side of the chambers were removed, and the chambers were subsequently fixed in 4% phosphate-buffered neutral formalin for 30 min. After 1% crystal violet staining, FBS washing and air drying, the remaining cells were photographed and counted to evaluate the migration and invasion abilities.

### *In vivo* experiments

For the subcutaneous tumor model, a total of 5 × 10^6^ cells in 100 μL PBS were injected into the flanks of 4-week-old male BALB/c nude mice. The subcutaneous tumor size was recorded every 3 days, and the tumor volume was calculated as follows: [(length × width^2^)/2]. Approximately 4 weeks later, the mice were sacrificed, and the subcutaneous tumors were harvested, measured and fixed. For the metastasis model, a total of 1 × 10^6^ cells in 100 μL PBS were injected into 6-week-old male BALB/c nude mice via the tail vein. The mice were sacrificed after 8 weeks, and the lungs were collected and fixed. The sections were subjected to H&E staining. The above experiments were approved by the Institutional Animal Care and Use Committee of Shaoxing People's Hospital.

### Fluorescence *in situ* Hybridization (FISH) Assay

A total of 5 × 10^4^ cells were added to the cell climbing and cultured in 24-well plates for approximately 24 h. Then, the cells were fixed in 4% paraformaldehyde (DEPC-treated) for 20 min and used for further experiments according to the manufacturer's protocol. A LINC01535-specific probe and FISH kit were designed and provided by Servicebio Technology Co., Ltd. (Wuhan, China). DAPI was used to stain the cell nuclei, and photos were taken with a positive fluorescence microscope.

### Luciferase reporter analysis

The wild-type (WT) or mutated (MUT) 3' UTR sequences of LINC01535/VASP containing the putative binding sites of miR-214-3p were inserted into the pGL3 plasmid by Genescript (Nanjing, China). The miR-214-3p mimic or NC was cotransfected into cells using Lipofectamine 3000 (Invitrogen). The luciferase activities were determined with the Dual-Luciferase Reporter Assay System (Promega, USA).

### Western blot

The total protein of HCC tissues and cells was obtained by precooled radioimmunoprecipitation assay (RIPA) buffer containing protease and phosphatase inhibitor cocktails. A bicinchoninic acid (BCA) protein assay kit (Beyotime, Nantong, China) was used to detect the protein concentrations. The protein was separated using sodium dodecyl sulfate-polyacrylamide gel electrophoresis (SDS-PAGE) and then transferred to polyvinylidene fluoride (PVDF) membranes (Merck Millipore, Burlington, MA, USA). After being blocked with skim milk, the PVDF membranes were incubated with specific primary antibodies overnight at 4 ℃. The membranes were washed with Tris-buffered saline-Tween (TBST) 3 times and then incubated with secondary antibody for 2 h at room temperature. After washing with TBST, the proteins on the membranes were visualized using Super ECL Detection Reagent (Yeasen, Shanghai, China) and Image Lab software (Bio-Rad, Hercules, CA, USA).

### Statistical analysis

The data analysis was performed using GraphPad Prism version 7.0 software (La Jolla, CA, USA) and SPSS version 18.0 software (Chicago, IL, USA). Two-tailed Student's t-test was used to evaluate the differences between two groups. The relationship between LINC01535 expression and clinical parameters was determined by the chi-squared test. The Kaplan-Meier test was used to assess survival curves, and the significant differences between survival curves were determined via the log-rank test. p < 0.05 indicates statistically significant differences.

## Results

### LINC01535 is upregulated in HCC and associated with poor overall survival

**Figure 1:**
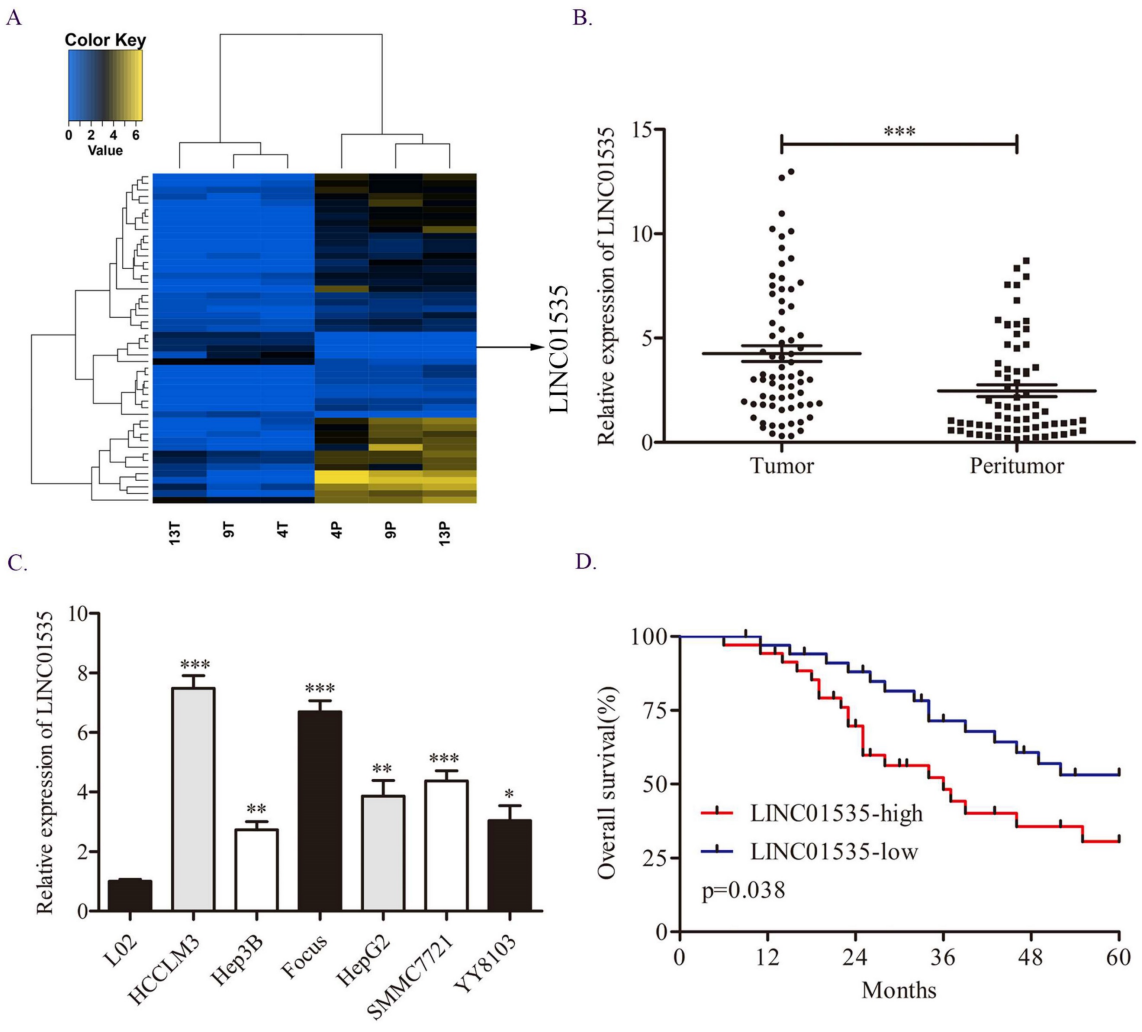
Overexpression of LINC01535 in HCC. A. The heat map of lncRNA sequencing. B. The expression of LINC01535 in 70 pairs of HCC samples and adjacent noncancerous tissues was analyzed by qRT-PCR. C. LINC01535 expression levels in HCC cells and the immortalized human L02 hepatocyte cell line were detected using qRT-PCR. D. The overall survival (OS) of 70 patients with HCC classified according to their relative LINC01535 expression level was analyzed via Kaplan-Meier analyses. (* P<0.05, ** P<0.01, *** P<0.001).

First, we explored the difference in lncRNA expression profiles in 3 pairs of HCC tissues via RNA sequencing assay. We noticed LINC01535, which was one of the top50 differentially expressed lncRNAs (Fig. 1A). Moreover, previous studies have shown that LINC01535 is upregulated in cervical cancer, osteosarcoma and esophageal squamous cell cancer and promotes tumor progression. However, the expression and function of LINC01535 in HCC has not been explored. qRT-PCR in 70 pairs of HCC tissues revealed that LINC01535 was overexpressed in HCC tissues compared to matched adjacent normal tissues (Fig. 1B). The same trend was observed in HCC cell lines (Fig. 1C). The clinicopathological analysis showed a close association among LINC01535 expression and tumor size and microvascular invasion (Table 1). Kaplan-Meier and log-rank analyses revealed that HCC patients with higher LINC01535 expression had shorter overall survival (OS) (Fig. 1D). The above data indicated that LINC01535 was overexpressed in HCC and might be associated with the progression of HCC.

### Downregulation of LINC01535 expression attenuates HCC cell proliferation and metastasis *in vitro*

**Figure 2:**
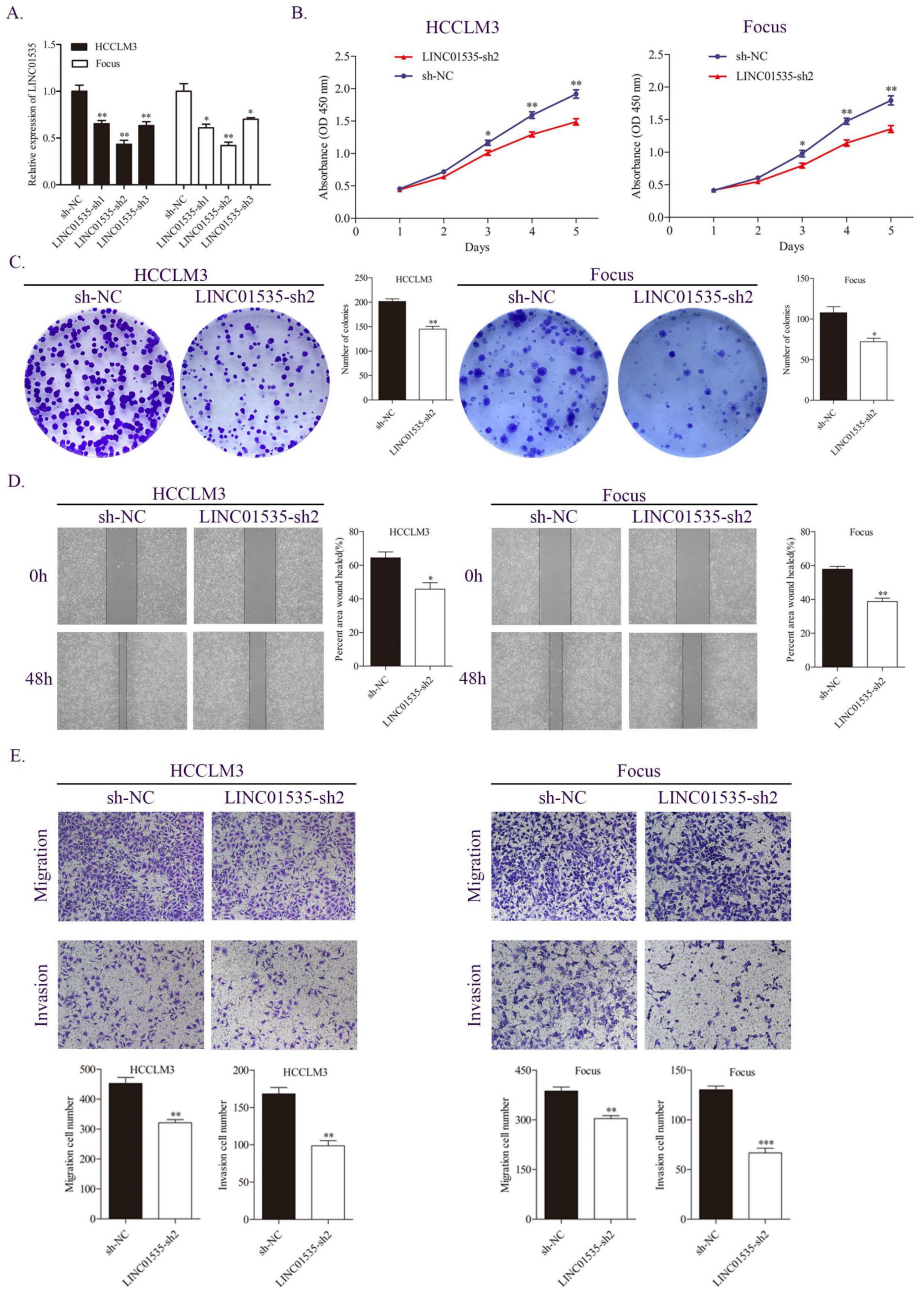
LINC01535 knockdown suppressed HCC cell proliferation, migration and invasion. A. The expression level of LINC01535 was detected by qRT-PCR after shRNA transfection. B. Growth curves based on the CCK-8 assay results of Focus and HCCLM3 cells with LINC01535 silencing. C. The number of colonies was reduced after LINC01535 downregulation in HCCLM3 and Focus cells. D. A wound healing assay was conducted to measure the migration ability of HCCLM3 and Focus cells after different treatments. E. Transwell assays suggested that LINC01535 knockdown inhibited HCC cell migration and invasion. (* P<0.05, ** P<0.01, *** P<0.001).

Based on the expression level of LINC01535 in HCC cell lines, we selected HCCLM3 and Focus to construct stable LINC01535 knockdown cell lines. The qRT-PCR results revealed that LINC01535-sh2 showed the best knockdown efficiency (Fig. 2A). CCK-8 and colony formation assays were performed to observe cell reproductive capacity *in vitro*. The results indicated that downregulation of LINC01535 attenuated cell growth and colony formation (Fig. 2B, C). Furthermore, we selected the wound healing assay and Transwell assay to determine whether LINC01535 expression modulates HCC cell migration and invasion. Compared with the sh-NC groups, downregulation of LINC01535 suppressed the migration and invasion of both the HCCLM3 and Focus cell lines (Fig. 2D, E). Collectively, the above results suggested that the downregulation of LINC01535 expression attenuated HCC cell proliferation, colony formation, migration and invasion.

### Upregulation of LINC01535 expression promotes HCC cell proliferation and metastasis

**Figure 3:**
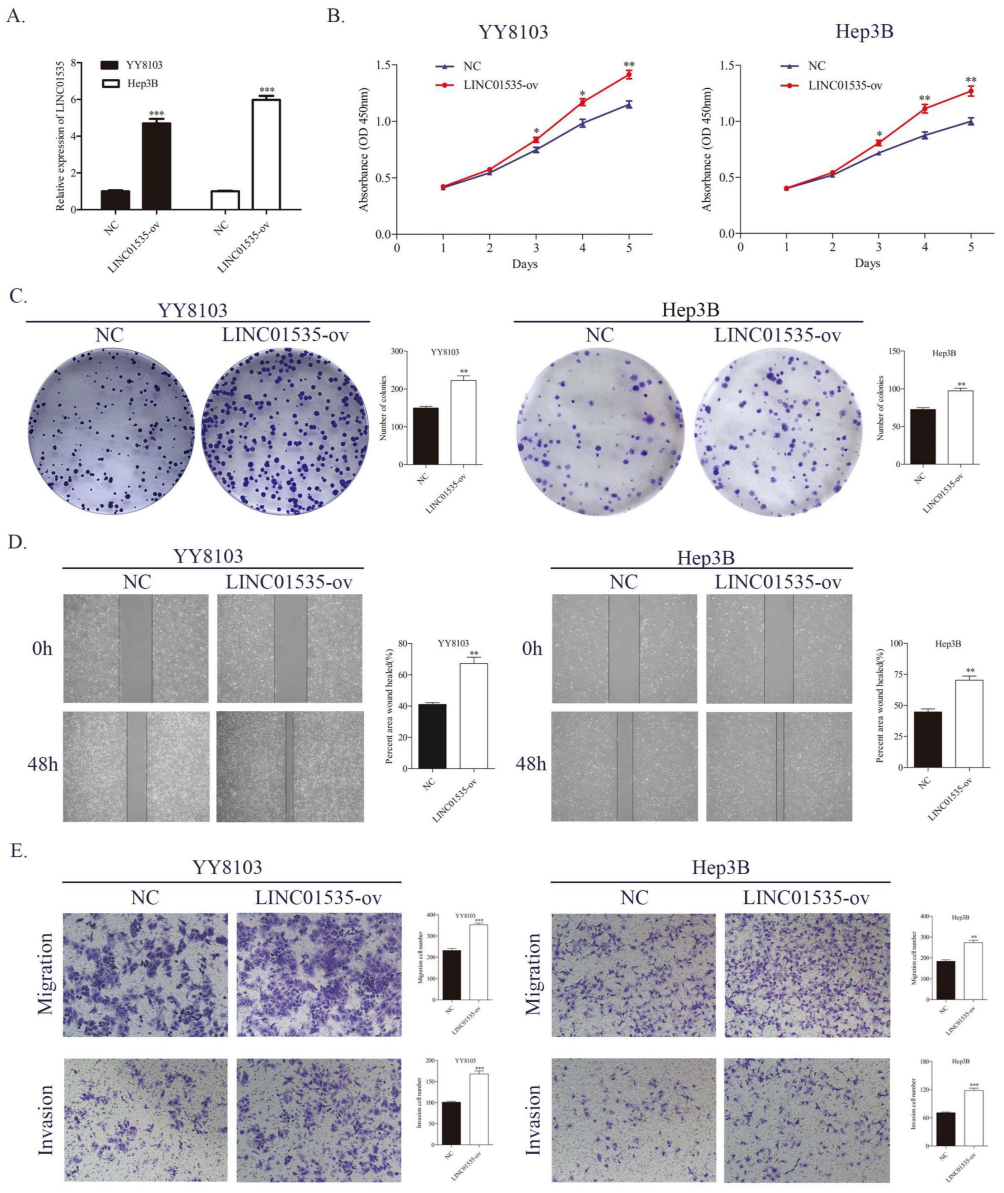
LINC01535 overexpression promoted HCC cell proliferation, migration and invasion. A. Assessment of the transfection efficiency of LINC01535 overexpression lentivirus. B-C. CCK-8 and colony formation assays were used to determine the growth and colony formation capacities of YY8103 and Hep3B cells transfected with either NC or LINC01535-ov. D-E. The wound healing assay and Transwell assays showed that HCC cells transfected with LINC01535 overexpression lentivirus exhibited stronger migration and invasion ability. (* P<0.05, ** P<0.01, *** P<0.001).

Lentivirus overexpressing of LINC01535 was constructed and transfected into YY8103 and Hep3B cells, and the overexpression efficiency was confirmed by qRT-PCR (Fig. 3A). The CCK-8 and colony formation assays showed that the overexpression of LINC01535 enhanced the proliferative and colony formation capacities of YY8103 and Hep3B cells (Fig. 3B, C). Similar results were seen in wound healing and Transwell assays, and LINC01535 upregulation also facilitated cell migration and invasion abilities (Fig. 3D, E). These data suggested that LINC01535 could promote HCC cell proliferation migration and invasion *in vitro*.

### LINC01535 promotes HCC cell proliferation and metastasis *in vivo*

**Figure 4:**
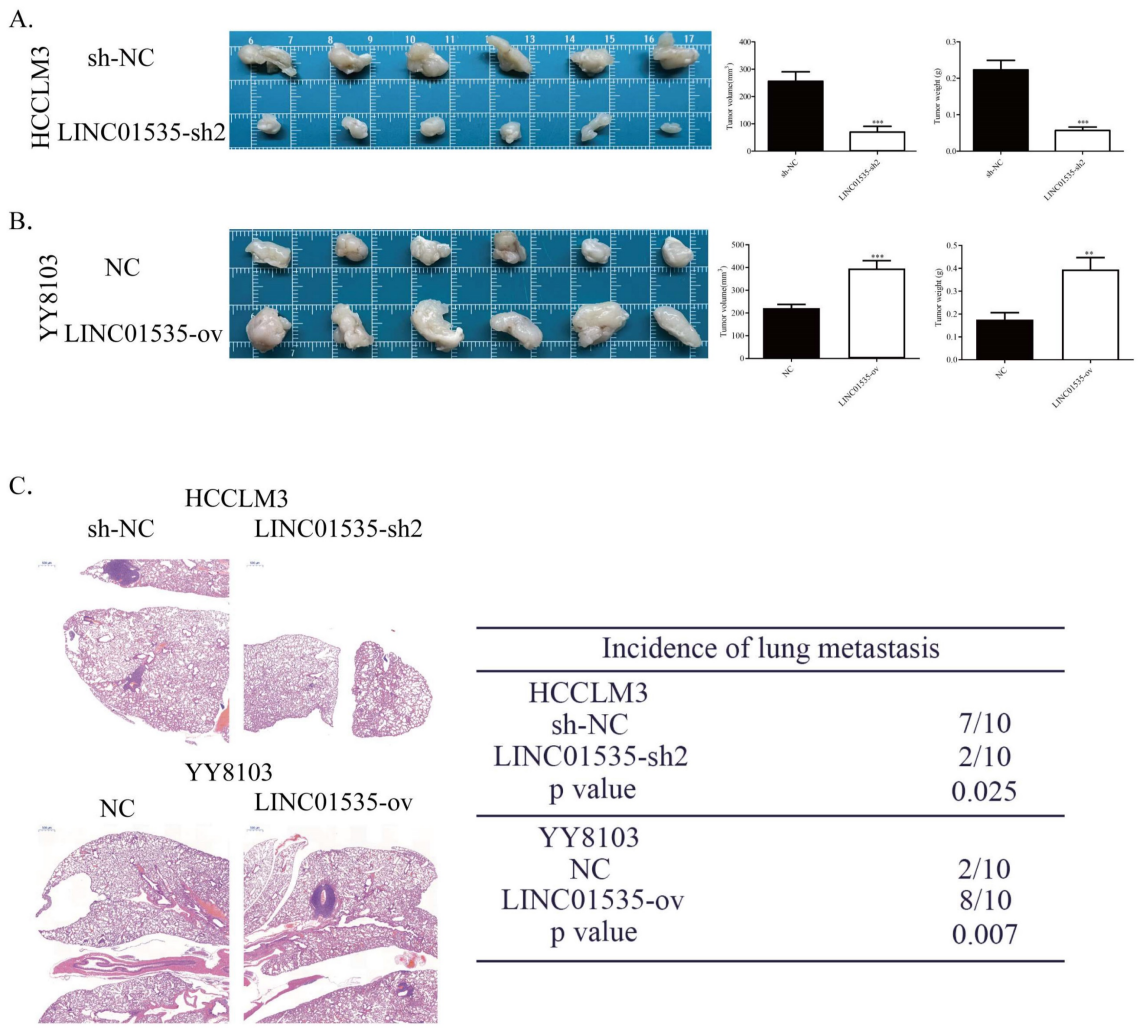
LINC01535 increased HCC cell growth and metastasis in vivo. A. Images of subcutaneous tumor tissues formed by HCCLM3 cells transfected with LINC01535-sh2 or sh-NC. The tumor volume and weight were recorded and analyzed. B. Images of subcutaneous tumor tissues formed by YY8103 cells transfected with LINC01535-ov or NC. The tumor volume and weight were recorded and analyzed. C. HE staining of lung tissue in different treatment groups (right, 20x) and analysis of the incidence of lung metastasis (left). (*** P<0.001).

To explore the effect of LINC01535 *in vivo*, we generated subcutaneous tumor and pulmonary metastasis mouse models via tail vein injection. As shown in Fig. 4A, LINC01535 knockdown suppressed tumor growth, while LINC01535 overexpression accelerated tumor growth (Fig. 4B). Consistent with the *in vitro* results, LINC01535 downregulation attenuated lung metastasis, while LINC01535 upregulation enhanced lung metastasis compared to that of the controls (Fig. 4C). *In vivo* results further confirmed that LINC01535 acts as an oncogene in HCC and promotes HCC cell proliferation and metastasis.

### miR-214-3p is a direct target of LINC01535 in HCC

**Figure 5:**
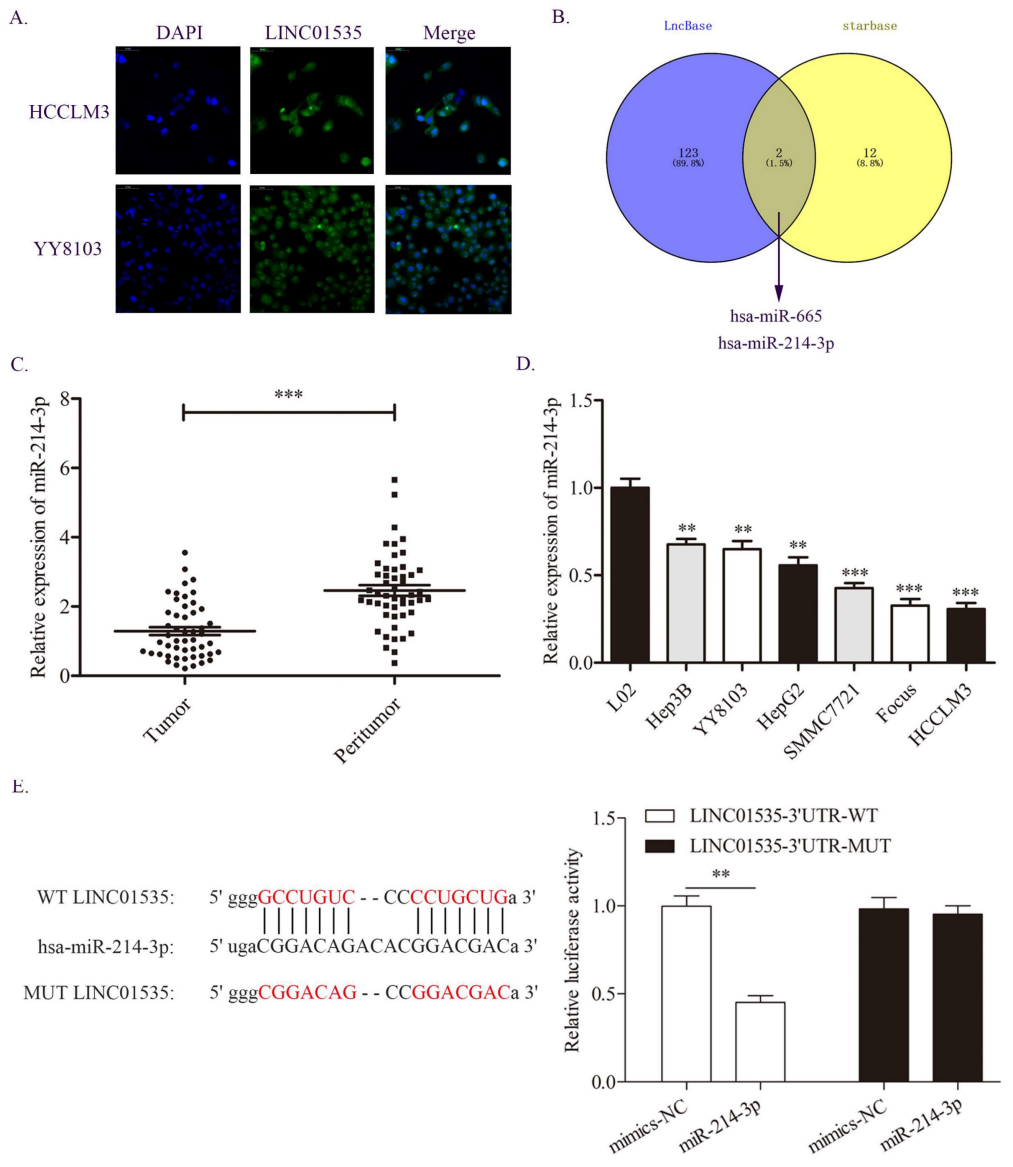
miR-214-3p was demonstrated to be a direct target of LINC01535. A. The distribution of LINC01535 in HCC cells was detected by FISH assay. B. Bioinformatics analysis of different databases suggested that miR-214-3p was the target of LINC01535. C-D. The expression level of miR-214-3p was detected in HCC tissues and cell lines. E. A dual-luciferase assay was conducted to assess the direct interaction of LINC01535 and miR-214-3p. (** P<0.01, *** P<0.001).

Because lncRNAs in different locations may function in different ways, we first detected the distribution of LINC01535 in HCC cells. The FISH assay results showed that LINC01535 was distributed in both the nucleus and cytoplasm of HCCLM3 and YY8103 cells (Fig. 5A). The distribution of LINC01535 in the cytoplasm suggests that LINC01535 may function by sponging miRNAs. We then carried out bioinformatics analysis from two databases, LncBase and starBase, to predict the potential targets of LINC01535. As shown in Fig. 5B, miR-665 and miR-214-3p were predicted to have putative with LINC01535 binding sites. For the two miRNAs, only miR-214-3p was reported to be involved in HCC progression. The qRT-PCR data revealed lower expression of miR-214-3p in HCC tissues and cell lines (Fig. 5C, D). To further confirm whether LINC01535 directly interacts with miR-214-3p, we conducted luciferase reporter analysis. Luciferase activity was decreased in HCCLM3 cells cotransfected with LINC01535-3' UTR-WT and miR-214-3p mimic (Fig. 5E). The results suggested that LINC01535 could act as a ceRNA by directly sponging miR-214-3p.

### LINC01535 promotes HCC progression by sponging miR-214-3p

**Figure 6:**
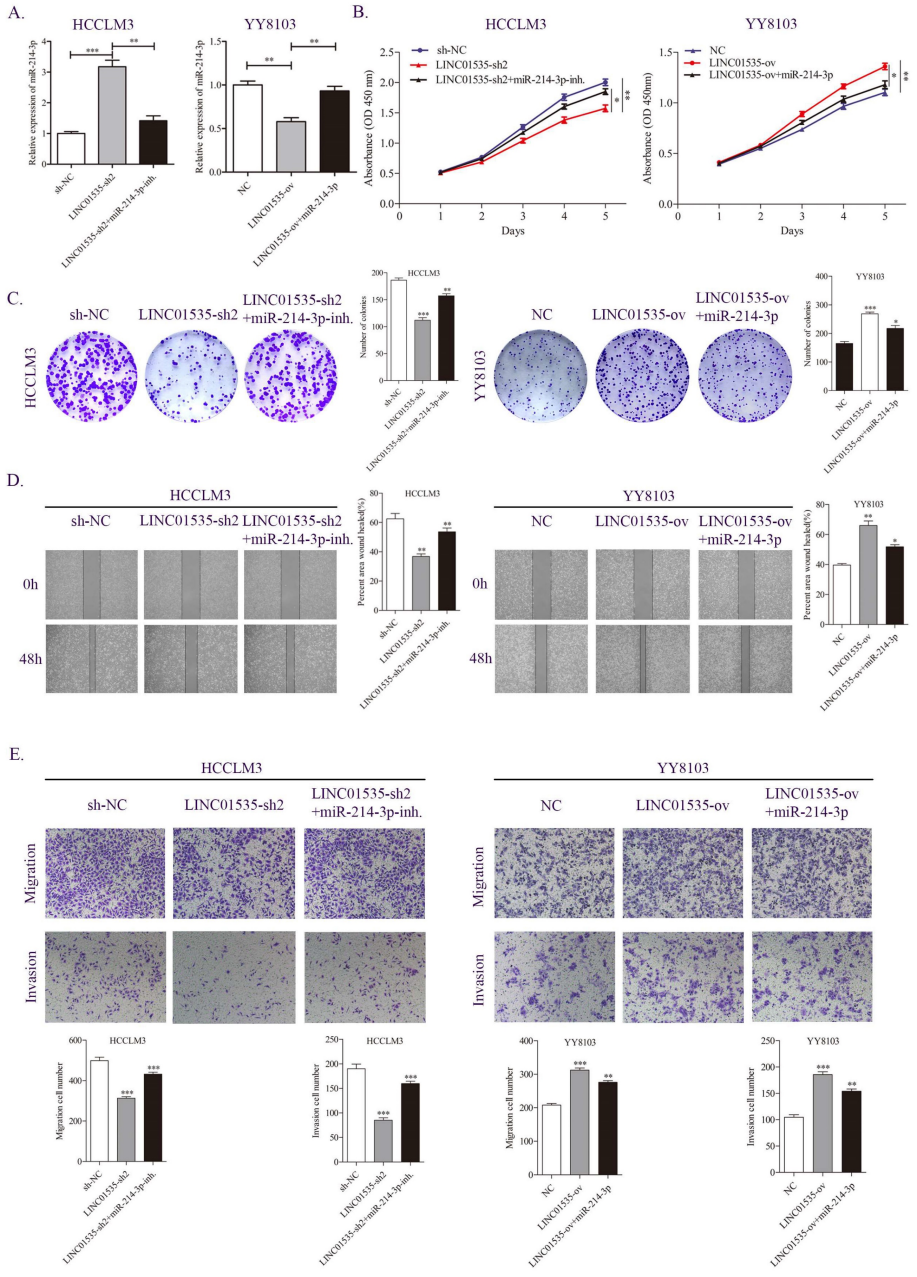
miR-214-3p partially reversed the effect of LINC01535 in HCC cells. A. The expression level of miR-214-3p in different groups. B-C. CCK-8 and colony formation assays were performed to determine the growth and colony forming abilities of the indicated groups. D-E. Wound healing and Transwell assays revealed the migration and invasion capacities of HCC cells after different treatments.

We further examined whether LINC01535 promotes HCC proliferation and migration through miR-214-3p. As shown in Fig. 6A, knockdown of LINC01535 increased, while overexpression of LINC01535 decreased the expression of miR-214-3p. The CCK-8 assay revealed that the inhibitory effect of LINC01535-sh2 on HCCLM3 cell growth could be partially restored by the miR-214-3p inhibitor, and the promotive effect of LINC01535 overexpression on YY8103 cell growth could be partially restored by cotransfection of miR-214-3p mimic (Fig. 6B). Similar results were obtained in the colony formation assay: LINC01535-sh2 reduced the colony formation activity of HCCLM3 cells, and LINC01535 overexpression enhanced the colony formation activity of YY8103. The effects of LINC01535 changes were reversed by simultaneous cotransfection of miR-214-3p inhibitor or mimic (Fig. 6C). Wound healing experiments showed that the miR-214-3p inhibitor strengthened, while miR-214-3p mimic reduced, the migratory ability of HCC cells caused by LINC01535 (Fig. 6D). The subsequent Transwell assays showed that HCCLM3 cells cotransfected with LINC01535-sh2 and miR-214-3p inhibitor exhibited higher metastatic and invasive capacities than cells transfected with LINC01535-sh2. However, the capacities of YY8103 cells were decreased in the LINC01535-ov+miR-214-3p group compared with the LINC01535-ov group (Fig. 6E). All the results indicated that miR-214-3p could partially reverse the effect of LINC01535 in HCC cells and that LINC01535 acts as a molecular sponge for miR-214-3p in HCC cells.

### VASP is a novel target of miR-214-3p in HCC cells

**Figure 7:**
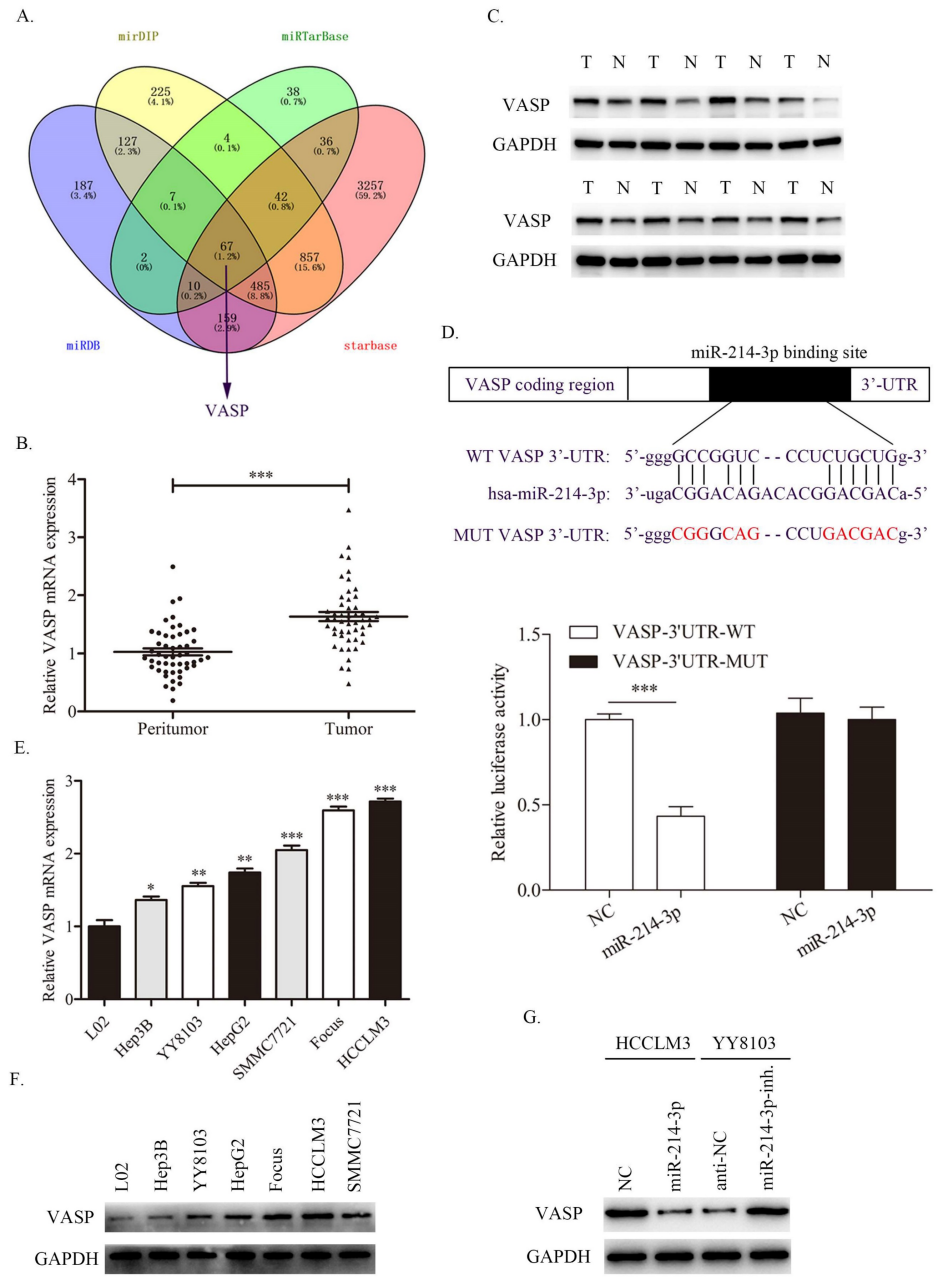
miR-214-3p directly targets and regulates VASP expression in HCC. A. Different online databases were used to predict miR-214-3p binding target genes. B-C. The mRNA and protein levels of VASP in HCC samples were detected by qRT-PCR and western blot. D. A dual-luciferase assay was performed to verify the binding effect between the VASP 3′UTR and miR-214-3p. E-F. The mRNA and protein expression levels of VASP in HCC cell lines. G. Western blot analysis revealed that miR-214-3p overexpression suppressed, while miR-214-3p downregulation promoted the expression of VASP in HCC cells. (* P<0.05, ** P<0.01, *** P<0.001).

To investigate the potential target of miR-214-3p in HCC, we searched four publicly available databases, including mirDIP, miRTarBase, miRDB and starBase. Interestingly, VASP was identified to have conjectural miR-214-3p binding sites (Fig. 7A). Previous studies have confirmed that VASP promotes HCC progression as an oncogene in HCC. First, we detected the expression level of VASP in HCC samples, and the qRT-PCR and western blot results revealed that both the mRNA and protein of VASP were upregulated in HCC tissues compared with adjacent normal tissues (Fig. 7B, 7C).

Consistently, the dual-luciferase assay showed that miR-214-3p overexpression suppressed the luciferase activity of the wild-type (WT) VASP 3'UTR but not the mutant (MUT) VASP 3'UTR (Fig. 7D). Next, the expression level of VASP in HCC cell lines was also measured, as shown in Fig. 7E and 7F. Consistent with the results in HCC samples, VASP was overexpressed in HCC cell lines compared with immortalized the human L02 hepatocyte cell line. Moreover, VASP was decreased in HCCLM3 cells transfected with miR-214-3p mimic and increased in YY8103 cells transfected with miR-214-3p inhibitor (Fig. 7G). The results confirmed that VASP is a direct target of miR-214-3p and is negative regulated by miR-214-3p.

### Alteration of VASP expression partially reverses the biological functions of miR-214-3p in HCC cells

**Figure 8:**
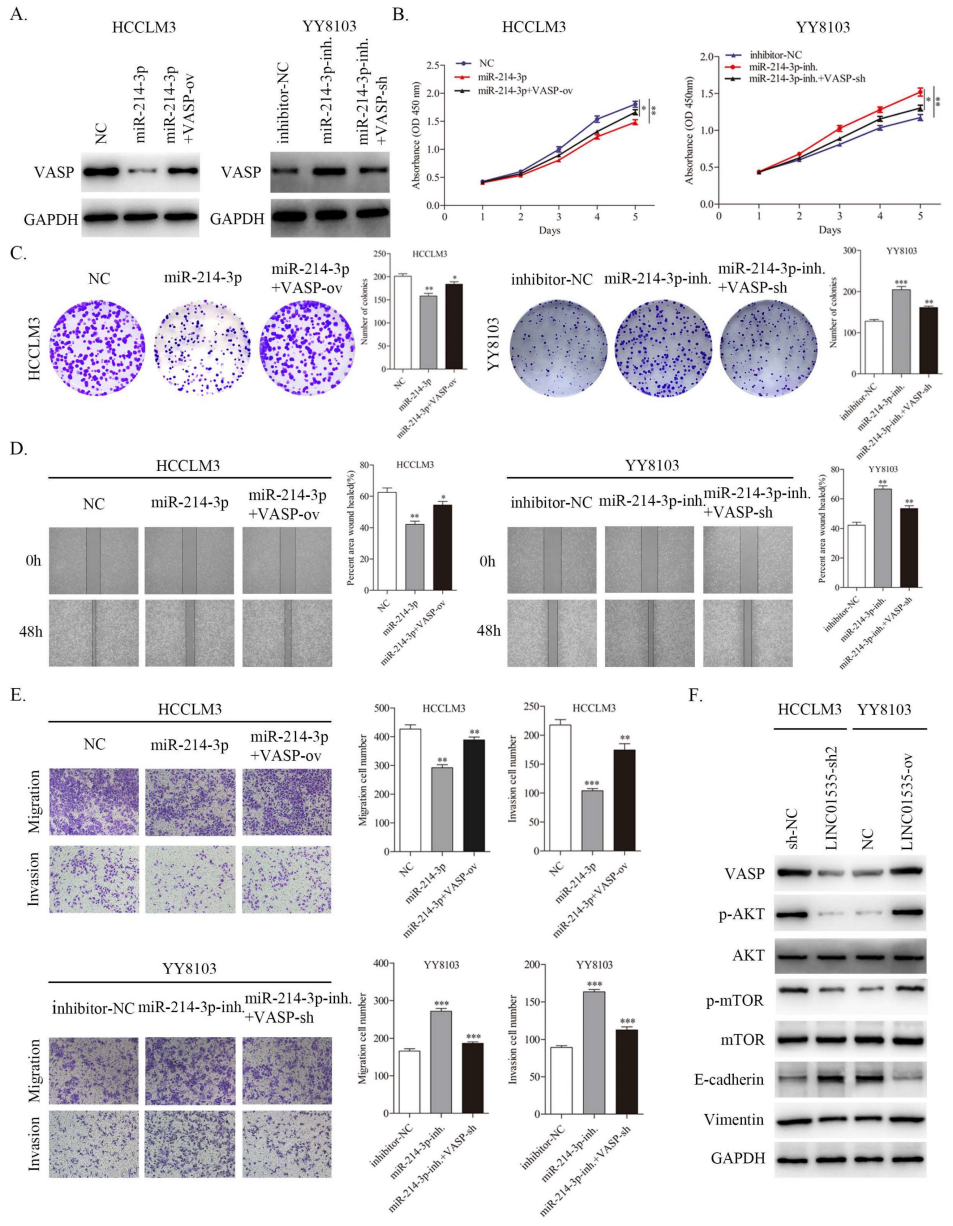
VASP partially reversed the function of miRNA-214-3p and LINC01535 regulated PI3K/Akt signaling and EMT via VASP. A. The expression of VASP in HCCLM3 and YY8103 cells subjected to different treatments. B-C. The growth and colony formation capacities of the indicated groups were detected through CCK-8 and colony formation assays. D-E. Wound healing and Transwell assays demonstrated that miR-214-3p impaired the migration and invasion of HCC cells, while VASP reversed the effects caused by miR-214-3p. F. Knocking down LINC01535 upregulated E-cadherin and downregulated VASP, p-AKT, p-mTOR and Vimentin proteins in HCCLM3 cells, while the opposite changes were observed in YY8103 cells overexpressing LINC01535. (* P<0.05, ** P<0.01, *** P<0.001).

To further confirm that VASP contributed to the function of miR-214-3p in regulating the proliferation and metastasis of HCC, we constructed different treatment groups of HCC cells, and the results were detected by western blot. As shown in Fig. 8A, miR-214-3p mimic downregulated the expression level of VASP, and cotransfection with VASP ov. partially restored the expression level of VASP in HCCLM3 cells. The miR-214-3p inhibitor upregulated the expression level of VASP, and cotransfection of VASP-sh also partially restored the expression level of VASP in YY8103 cells. CCK-8 and colony formation assays suggested that miR-214 overexpression suppressed cell growth, while VASP overexpression rescued the capacity of HCCLM3 cells. Moreover, the miR-214-3p inhibitor enhanced cell growth, and VASP-sh also attenuated the effect of the miR-214-3p inhibitor in YY8103 cells (Fig. 8B, C). Similar results were obtained in wound healing and transwell assays. The miR-214-3p inhibitor strengthened, while miR-214-3p mimic suppressed, HCC cell migration and invasion, and VASP partially reversed the functions of miR-214-3p (Fig. 8D, E). Based on these results, miR-214-3p regulates HCC proliferation and metastasis by targeting VASP.

### LINC01535 plays a regulatory role in the PI3K/AKT signaling pathway and induces EMT progression via VASP in HCC

Since VASP has been reported to activate PI3K/AKT signaling and promote EMT progression in HCC 19, we further invastigated whether LINC01535 could influence PI3K/AKT signaling and EMT via regulating VASP. The western blot results showed that LINC01535 knockdown decreased the protein expression levels of VASP, p-AKT, p-mTOR, and Vimentin and increased the expression level of E-cadherin in HCCLM3 cells, while LINC01535 overexpression increased the protein expression levels of VASP, p-AKT, p-mTOR and Vimentin and decreased the expression level of E-cadherin in YY8103 cells (Fig. 8F). Together, these findings demonstrated that LINC01535 regulates PI3K/AKT signaling and EMT via VASP.

## Discussion

Looking for new and effective diagnostic and therapeutic targets will be helpful for improving the prognosis of patients with HCC 18. An increasing number of new transcripts are being discovered by transcriptome sequencing. Among them, lncRNAs have received increasing attention and been increasingly studied due to their essential roles in a variety of human tumors, including HCC 6, 20. Thus, we conducted RNA sequencing to explore the differential expression profile of lncRNAs in HCC tissues. Interestingly, we found that LINC01535, which is one of the most distinct lncRNAs, acts as an oncogene in osteosarcoma 11, esophageal squamous cell cancer 12 and cervical cancer 10. Further qRT-PCR data confirmed the upregulated expression of LINC01535 in HCC samples and cell lines. Moreover, the high level of LINC01535 was strongly correlated with tumor size, microvascular invasion and OS. The results indicated that LINC01535 may also act as an oncogene in HCC. Next, loss-of-function and gain-of-function experiments indicated that LINC01535 knockdown inhibited, while LINC01535 overexpression promoted HCC cell growth, colony formation, migration and invasion both *in vitro* and *in vivo*. Our results confirmed that LINC01535 promotes the progression of HCC.

One of the most recognized mechanisms of action of lncRNAs is that lncRNA act as ceRNAs to competitively sponge miRNAs, resulting in reduced binding of miRNAs to target genes and thereby regulating the expression of target genes 21. Liu *et al.* reported that lncRNA HOTAIR promoted gastric cancer growth and metastasis by sponging miR-331-3p 22. Li *et al.* discovered that lncRNA-SHG7 acts as an oncogene in colorectal cancer via the SNHG7/miR-34a/GALNT7 axis 23. SNHG6-003, as a ceRNA, was found to be overexpressed and accelerate HCC progression 24. In this study, we first confirmed the distribution of LINC01535 in HCC cells through FISH assay. LINC01535 is mainly distributed in the cytoplasm, suggesting that it may act as a ceRNA. Bioinformatics analysis, luciferase reporter analysis and rescue experiments confirmed our hypothesis that LINC01535 acts as a ceRNA to inversely regulate the miR-214-3p levels and to weaken the suppressive effect of miR-214-3p on VASP.

miR-214-3p has been shown to play a tumor suppressive role in a variety of tumors 25-28. In HCC, miR-214-3p has also been reported to suppress tumor progression through different molecular mechanisms 29-33. Whether miR-214-3p regulates other target genes in HCC is worthy of further exploration. Consistent with a previous study, our results showed that miR-214-3p was downregulated in our HCC samples and cell lines, and miR-214-3p overexpression reduced HCC cell growth and metastasis. Furthermore, miR-214-3p was demonstrated to directly target VASP and negatively regulate its expression. Published studies have revealed that VASP is upregulated in cancers such as breast cancer 34, glioma 35 and HCC 36 and promotes the biological function of cancer cells by activating PI3K/AKT pathway 19, 37. Our studies demonstrated that LINC01535 overexpression upregulated VASP, resulting in activation of PI3K/AKT and promotion of EMT progression. The results indicated that LINC01535 acted as a competing endogenous RNA for miR-214-3p, thus increasing the expression of VASP.

In summary, we showed that LINC01535 is overexpressed in HCC and acts as an oncogene promote HCC proliferation and metastasis. Furthermore, LINC01535 competitively sponges miR-214-3p, and subsequently reduces the suppressive effect of miR-214-3p on VASP, resulting in activation of the PI3K/AKT pathway and acceleration of EMT progression. The ceRNA network involving LINC01535 may provide new targets for the diagnosis and treatment of HCC.

### Strengths related to the current study

In this study, we identified the upregulation of LINC01535 expression in HCC for the first time using RNA sequencing and qRT-PCR. Furthermore, a series of *in vitro* and *in vivo* experiments confirmed that LINC01535 promotes the proliferation and metastasis of HCC cells through the LINC01535/miR-214-3p/VASP axis. This discovery of the function of LINC01535 and this novel regulatory mechanism provide new possibilities for diagnosing and treating HCC.

### Limitations

Whether LIN01535 regulates HCC progression through other molecular mechanisms and the specific molecular mechanisms of VASP regulating PI3K/AKT axis still need to be further studied.

### Recommendation and future perspectives

We plan to investigate the therapeutic potential of LINC01535 using small molecule drugs that target the LINC01535/miR-214-3p/VASP axis, as well as biomaterials or adeno-associated virus vectors carrying LINC01535 siRNA. Furthermore, consider vitamin E as a prophylactic measure for inflammation and oxidative stress-related diseases, as it may be a better option than vitamin D 38. Vitamin E has shown potential for repurposing in cancer treatment 39. Therefore, exploring therapeutic approaches, such as utilizing vitamin E as an adjuvant therapy, can contribute to the development of comprehensive and effective strategies for managing HCC in the future.

## Figures and Tables

**Table 1:** Correlation between LINC01535 expression and clinicopathological features.

Clinicopathological features		All cases	LINC01535		*p* value
			High expression	Low expression	
Age (years)	>60	39	20	19	0.810
	≤60	31	15	16	
Gender	Female Male	28 42	15 20	13 22	0.626
HBV	Negative	9	3	6	0.284
	Positive	61	32	29	
Tumor multiplicity	Single	43	18	25	0.086
	Multiple	27	17	10	
Tumor size (cm)	≤5	42	16	26	**0.015**
	>5	28	19	9	
α-fetoprotein (ng/ml)	≤200	27	12	15	0.461
	>200	43	23	20	
TNM stage	I	40	16	24	0.053
	II-III	30	19	11	
Microvascular invasion	Yes	15	11	4	**0.041**
	No	55	24	31	

HBV, hepatitis B virus; TNM, tumor-node-metastasis. The bold number means statistically significant.

## Abbreviations

LncRNAs: long noncoding RNAs
HCC: hepatocellular carcinoma
FISH: Fluorescence *in situ* hybridization
qRT-PCR: quantitative real-time polymerase chain reaction
miR-214-3p: microRNA-214-3p
EMT: epithelial-to-mesenchymal transition
ceRNA: competing endogenous RNA
MREs: miRNA response elements
3'-UTRs: 3'-untranslated regions
DMEM: Dulbecco's modified Eagle's medium
FBS: fetal bovine serum
shRNA: small hairpin RNA
NC: negative controls
miR-214-3p-inh.: miR-214-3p inhibitor
IHC: Immunohistochemistry
CCK-8: counting Kit-8
WT: wild-type
MUT: mutated
RIPA: radioimmunoprecipitation assay
BCA: bicinchoninic acid
SDS-PAGE: sodium dodecyl sulfate-polyacrylamide gel electrophoresis
PVDF: polyvinylidene fluoride
TBST: Tris-buffered saline-Tween
OS: overall survival. TNM: tumor-node-metastasis
HE: Hematoxylin and eosin
